# Integrating local and global information to identify influential nodes in complex networks

**DOI:** 10.1038/s41598-023-37570-7

**Published:** 2023-07-14

**Authors:** Mohd Fariduddin Mukhtar, Zuraida Abal Abas, Azhari Samsu Baharuddin, Mohd Natashah Norizan, Wan Farah Wani Wan Fakhruddin, Wakisaka Minato, Amir Hamzah Abdul Rasib, Zaheera Zainal Abidin, Ahmad Fadzli Nizam Abdul Rahman, Siti Haryanti Hairol Anuar

**Affiliations:** 1 Universiti Teknikal Malaysia Melaka, 76100 Durian Tunggal, Malaysia; 2 Universiti Putra Malaysia (UPM), 43400 Serdang, Selangor Darul Ehsan Malaysia; 3 Universiti Malaysia Perlis, 02600 Kampung Ulu Pauh, Perlis Malaysia; 4 Universiti Teknologi Malaysia, 81310 Johor Bahru, Johor Malaysia; 5 Fukuoka Women’s University, Fukuoka, 813-8529 Japan

**Keywords:** Computational science, Computer science

## Abstract

Centrality analysis is a crucial tool for understanding the role of nodes in a network, but it is unclear how different centrality measures provide much unique information. To improve the identification of influential nodes in a network, we propose a new method called Hybrid-GSM (H-GSM) that combines the K-shell decomposition approach and Degree Centrality. H-GSM characterizes the impact of nodes more precisely than the Global Structure Model (GSM), which cannot distinguish the importance of each node. We evaluate the performance of H-GSM using the SIR model to simulate the propagation process of six real-world networks. Our method outperforms other approaches regarding computational complexity, node discrimination, and accuracy. Our findings demonstrate the proposed H-GSM as an effective method for identifying influential nodes in complex networks.

## Introduction

Complex networks refer to intricate systems composed of interconnected elements, such as nodes and edges, where the interactions between these elements exhibit non-trivial patterns^1^. To comprehensively analyze complex networks, researchers often explore them from four essential perspectives: path analysis, connectivity, community, and centrality. These analytical approaches shed light on the intricate pathways, structural connections, cohesive groups, and influential nodes within the network, enabling a holistic understanding of its dynamics and characteristics^2^. One of the most essential and challenging research challenges in network science is determining and prioritizing the most important nodes. The process of discovering and ranking the most influential nodes (INs) is critical for gaining a thorough view of a network's structure and operation ^3^. Several centrality metrics have been presented throughout the years to capture a network's rank based on node degree and importance in the network's structure^4–7^. It is considered that the efficiency of a centrality measure in finding key nodes is dependent on its topological significance^8^. Currently, a website (http://www.centiserver.org)^9^ has documented that there were approximately 403 centrality indices, providing a comprehensive resource for network analysis. However, despite this vast compilation, the exploration of identifying the most (INs) within complex networks remains an ongoing pursuit.

In latest years, methodologies for locating prominent nodes have gotten more targeted, relying only on global or local data. For example, K-shell decomposition (Ks)^10,11^ and the Degree Centrality (DC)^12^ approach is two of the most thoroughly explored interpretations of global and local information, respectively. Because of their simplicity, these two techniques have achieved broad use in networks of all sizes. However, Ks and DC have limits in determining the relative significance of nodes in a network.

In Ks, first and foremost, previous knowledge about the value of k is required, which may not be easily accessible^13^. Second, since the Ks is based on local connection^14^, it may not be useful in recognising the hierarchical structure of networks. Finally, it may not correctly represent the underlying structural aspects of the network since it may not capture the relevance of nodes that operate as bridges between various levels. It may be susceptible to the particular technique used to calculate the Ks, making it less accurate for comparing networks^15–17^.

On the other hand, DC is a standard network analysis metric that rates a node's relevance based on the number of edges (links) it has in the network. One issue is that it does not consider how excellent or crucial the relationships are^18–20^. Nodes with a high degree of centrality may have numerous connections, but those connections may be with nodes that are not central or significant in their own right^21^. Moreover, degree centrality does not take into account a node's structural location in the network^22^, which might influence its relevance. Lastly, degree centrality is less effective in directed networks with incoming and outward edges that need differentiation between in-degree and out-degree centrality measurements^20,22,23^. Overall, degree centrality is a valuable metric for detecting INs; however, it should be used in conjunction with other measures that capture other characteristics of a node's significance in a network^14,21,22^.

Other common centrality methods, such as betweenness centrality (BC)^24^ and closeness centrality (CC)^25^, which estimate node impact based on global network information and may give higher ranking results, have a significant processing cost^26,27^. Geographical information, both local and global, may have a substantial influence on the power of INs in a network.

^17,18,28–31^Researchers have focused on local and global network information to solve the problem of identifying INs^17,18,28–30^. Unfortunately, past traditional identification approaches frequently missed critical information, failing to account for global and local network information simultaneously^7,31^. As a result, the outcomes are often skewed. Information about the neighbour nodes do increase the accuracy and correctness of a method^32^. Researchers discovered that combining both in a network improved the identification of influential nodes. This integration enhanced detection at both the local (in community or cluster) and global levels^7,33–35^. Taking the coreness and shortest distance between nodes into account might improve the discovery of INs.

Recently, an innovative approach is being introduces which is the Global Structure Model (GSM)^29^ and its improved version, IGSM^18^, to identify INs in these networks. These approaches apply local and global information, which are Ks and DC, respectively. Yet, one key weakness of both methodologies is their inability to quantify the significance of individual nodes, leaving a large vacuum in our knowledge of complex networks. As the need for more accurate and extensive network research grows, new approaches that may overcome this constraint and give deeper insights into the structure of complex networks must be developed.

The primary contributions of this paper lie in the development and application of the Hybrid Global Structure Model (H-GSM). The H-GSM algorithm addresses the deficiencies of current techniques by considering both local and global information of each node, resulting in a more comprehensive understanding of the overall structure of complex networks. Specifically, our contributions are as follows:

A) *Introducing a technique for identifying influential nodes*The H-GSM algorithm combines local information (DC) and global influence (Ks), overcoming the limitations of single-measure centrality approaches. By integrating local and global influences, our method significantly enhances the accuracy of identifying INs, surpassing traditional centrality measures such as DC, BC, PR, CC, GSM, and IGSM.
B) *High performance of the algorithm*Through extensive experiments conducted on six real complex networks, our technique demonstrates superior performance in identifying INs. The results surpass those obtained using the widely used SIR model and Kendall's correlation coefficient, showcasing the effectiveness and reliability of the H-GSM algorithm.
C) *Superior scalability compared to other algorithms*The H-GSM algorithm exhibits excellent scalability, making it suitable for larger networks. Its low computational complexity and straightforward implementation further enhance its applicability, providing a practical and efficient solution for analyzing complex network structures.

Overall, the H-GSM algorithm contributes to the advancement of network analysis by offering a novel approach that combines local and global influences, outperforming existing centrality measures, and providing superior scalability for large-scale network analysis.

The rest of this paper is organised as follows: In the section titled "Method", a brief introduction of numerous baseline approaches and the suggested H-GSM method are explained in detail. Next, a total of six actual networks data from the real-world case study have been adopted and used to validate the proposed method, which are described in the sections titled "Datasets and evaluation criteria" and "Results and discussions" respectively. This study's findings and recommendations for the future work are presented in the final section, titled "Conclusion and Future Recommendations".

## Methods

### Background analysis

Suppose a network is denoted as $G = \left( {V,E} \right)$ where *V* is the set of nodes and *E* represents the edges. If there is an edge between node *i* and node* j*, then $a_{ij} = 1$ they are directly connected, while if there is no edge, then $a_{ij} = 0$ they are not directly connected. The total number of nodes in the network is denoted as *n*. The indices that use in this study are introduced in this section.

### Degree centrality (DC)

The number of nodes close to or directly linked to a node is denoted by DC, which is the most basic form of centrality. DC reflects on node information at the most local level, which is straightforward and intuitive. The higher the degree, the bigger the effect of the node. A node's degree centrality formula is as follows:

1 $${\text{DC}}(i) = \sum\limits_{j = 1}^{n} {a_{ij} }$$

### Betweenness centrality (BC)

The BC of a node is the ratio of the shortest pathways via the node to the total number of quickest routes. BC computes INs based on global data. A node with a high BC value serves an important function in linking various areas of the network. BC stands for

2 $${\text{BC}}(i) = \sum\limits_{j,k \ne 1}^{{}} {\frac{{g_{jk} (i)}}{{g_{jk} }}}$$

where $g_{jk}$ indicates the number of paths and $g_{jk} (i)$ represents the shortest paths between nodes $j$ and $k$ through a node $i$.

### Closeness centrality (CC)

CC also computes prominent nodes based on global data. CC indicates a node's proximity to all other nodes in the network. It uses the shortest distance ($d_{ij}$) between each pair of nodes to identify the influence of each node. CC of a node is defined as

3 $${\text{CC}}(i) = \frac{N - 1}{{\sum\limits_{j \ne 1} {d_{ij} } }}$$

### K-shell decomposition (Ks) method

Ks is one of the global centrality approaches for determining the core location of a network. Ks gives an index to each network node by deleting nodes repeatedly depending on their degree. Nodes with one connection are removed, and the network's degree value is recalculated. Stripping additional degree nodes continues until no more nodes can be stripped. A node with a higher Ks-value is more significant in the network and should be given more attention or consideration when interpreting the model or making choices based on its predictions. The Ks metric indicates that a cluster of nodes will exhibit comparable significance within a network^11^, yet it falls short in equitably distinguishing the nodes that possess greater influences.

### Global structure model (GSM)

GSM considers the node's self-influence and global influence. The influence of node *i* can be expressed as

4 $${\text{GSM}}(i) = {\text{SI}}(i){\kern 1pt} \; \times \;{\text{GI}}(i)\,{\kern 1pt} = e^{{{\raise0.7ex\hbox{${Ks(i)}$} \!\mathord{\left/ {\vphantom {{Ks(i)} n}}\right.\kern-0pt} \!\lower0.7ex\hbox{$n$}}}} \,\, \times \,\,\sum\limits_{i \ne j} {\frac{Ks(j)}{{d_{ij} }}}$$

where $Ks\left( i \right)$ and $Ks\left( j \right)$ denote the k-shell of node *i* and node *j,* respectively.

### Improved global structure model (IGSM)

IGSM is an improved GSM model considering DC instead of the Ks method.

5 $${\text{IGSM}}(i) = {\text{improved\_SI}}(i){\kern 1pt} \; \times \;{\text{improved\_GI}}(i)\,{\kern 1pt} = e^{{{\raise0.7ex\hbox{${DC(i)}$} \!\mathord{\left/ {\vphantom {{DC(i)} n}}\right.\kern-0pt} \!\lower0.7ex\hbox{$n$}}}} \,\, \times \,\,\sum\limits_{i \ne j} {\frac{DC(j)}{{d_{ij}^{{{\text{ceil}}\left( {{\text{log}}_{2} (ave\_DC)} \right)}} }}}$$

### Proposed method

The approach suggested in this study outperforms the GSM and IGSM methods already in use to identify INs in a network. The algorithm employs two indices: DC and Ks, and the suggested technique takes into account node position information. This is due to the fact that node placement is important in data distribution, and nodes in crucial places may have a stronger effect on the flow of information or resources within the network. The suggested technique offers a more complete approach to identifying INs in a network by combining these measurements and including location information.

To enhance the notion of node influence, we used the GSM's concept of self- and global impact, but applied it in a creative way. By increasing a node's DC by its Ks value, we established enhanced self-influence (iSI). This iSI factor was then used to calculate enhanced global impact (iGI), which takes into account all nodes that are directly or indirectly related to a node. The iGI factor is the sum of the neighbour ratios of the shortest route lengths for directed and undirected nodes with Ks and DC values. Its shortest route length is calculated by the average iSI value and is also referred to as information loss. In assessing node impact, the proposed H-GSM method takes into account both iSI and iGI parameters.

This suggested approach is significant because it can more accurately quantify how nodes in a network impact one another. Our strategy considers a node's local and global impacts, as well as how it affects other nodes in the network and the network as a whole. Consequently, including node position information aids in better understanding of data dispersion throughout the network. The new technique is likely to outperform current methods in terms of node determination, making it an important contribution to the area of network analysis. The complete equation is as follows:

6 $$iSI\left( i \right) = e^{{\frac{Ks(i)\; \times \;DC(i)}{N}}}$$

7 $$iGI\left( i \right) = \sum\limits_{i \ne j} {\frac{iSI(j)}{{d_{ij}^{{cell\left( {\log_{2} \left( {ave\_iSI} \right)} \right)}} }}}$$

8 $$\begin{gathered} {\text{H - GSM}}(v_{i} ) = iSI\left( i \right)\; \times \;iGI\left( i \right) \\ = e^{{\frac{Ks(i)\; \times \;DC(i)}{N}}} \; \times \;\sum\limits_{i \ne j} {\frac{{e^{{\frac{Ks(j)\; \times \;DC(j)}{N}}} }}{{d_{ij}^{{cell\left( {\log_{2} \left( {ave\_iSI} \right)} \right)}} }}} \\ \end{gathered}$$

### Computation process

**Figure 1:**
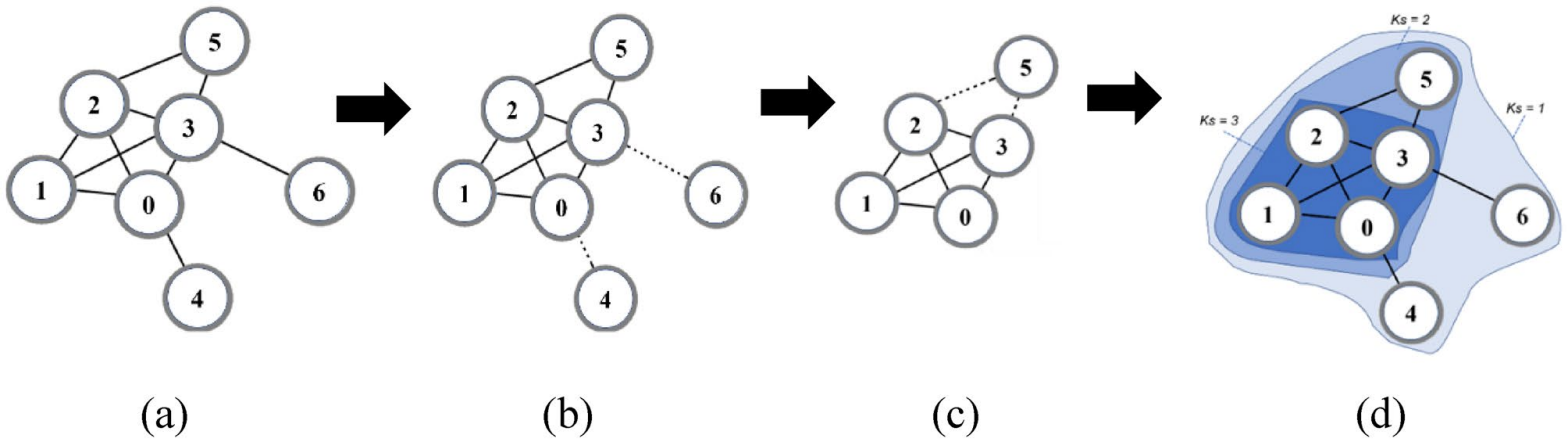
(**a**) A network with 7 nodes and 10 edges. (**b**) Removing of the most outer layer, which is node 4 and 6. (**c**) Removing of the next outer layer, which is node 5. (**d**) Summary of the Ks-territory of all the nodes in the network.

Figure 1 depicts a basic network with 7 nodes and 10 edges segregated by its k-shell territory to further clarify the specific calculation procedure of the H-GSM algorithm. As indicated in the network, we consider the H-GSM approach by using node 3 as an example of the targeted node, hence i = 3. Node 3 is positioned on the third layer, designated by Ks = 3, as are nodes 0, 1, and 2. In terms of DC value, node 3 obviously has five edges attached to it, resulting in DC-value = 5. We begin by computing the Ks, DC, and shortest distance between each node.

Step 1: Determine Ks and DC value.

$$Ks(3) = 3\quad \quad DC(3) = 5$$

Step 2: Calculate improved self-influence (iSI).

$$\begin{gathered} iSI(3) = e^{{\left[ {\frac{Ks(3) \times DC(3)}{n}} \right]}} \\ = e^{{\left[ {\frac{3 \times 5}{7}} \right]}} \\ = 8.523756 \\ \end{gathered}$$

Step 3: Calculate improved global influence (iGI).

$$\begin{gathered} iGI(3) = \sum\limits_{i \ne j} {\frac{kSI(j)}{{d_{ij}^{{cell\left( {\log_{2} \left( {kSI} \right)} \right)}} }}} \\ = \frac{kSI(0)}{{d_{ij}^{{cell\left( {\log_{2} \left( {ave\_kSI} \right)} \right)}} }} + \frac{kSI(1)}{{d_{ij}^{{cell\left( {\log_{2} \left( {ave\_kSI} \right)} \right)}} }} + \frac{kSI(2)}{{d_{ij}^{{cell\left( {\log_{2} \left( {ave\_kSI} \right)} \right)}} }} + \frac{kSI(4)}{{d_{ij}^{{cell\left( {\log_{2} \left( {ave\_kSI} \right)} \right)}} }} + \frac{kSI(5)}{{d_{ij}^{{cell\left( {\log_{2} \left( {ave\_kSI} \right)} \right)}} }} + \frac{kSI(6)}{{d_{ij}^{{cell\left( {\log_{2} \left( {ave\_kSI} \right)} \right)}} }} \\ = \frac{3.617251}{{1_{{}}^{{cell\left( {\log_{2} \left( {3.903478} \right)} \right)}} }} + \frac{5.552708}{{1_{{}}^{{cell\left( {\log_{2} \left( {3.903478} \right)} \right)}} }} + \frac{5.552708}{{1_{{}}^{{cell\left( {\log_{2} \left( {3.903478} \right)} \right)}} }} + \frac{1.153565}{{2_{{}}^{{cell\left( {\log_{2} \left( {3.903478} \right)} \right)}} }} + \frac{1.770795}{{1_{{}}^{{cell\left( {\log_{2} \left( {3.903478} \right)} \right)}} }} + \frac{1.153565}{{1_{ij}^{{cell\left( {\log_{2} \left( {3.903478} \right)} \right)}} }} \\ = 17.93542 \\ \end{gathered}$$

Step 4: Calculate node influence of H-GSM.

$$\begin{gathered} H - GSM(3) = iSI(3) \times iGI(3) \\ = 8.523756 \times 17.93542 \\ = 152.877133 \\ \end{gathered}$$

As illustrated in Fig. 1, Table 1 presents node rankings based on the implementation of the DC, BC, CC, Ks, GSM, IGSM, and H-GSM methodologies. Earlier works such as DC, KS, BC, and CC could only distinguish between six levels. Nonetheless, DC is better at level discrimination than KS and BC. The node rating for CC and GSM is the same. In finding the most INs in the network, both H-GSM and IGSM outperform standard GSM. For example, in GSM, rank 2 cannot tell which node is more significant between nodes 1 and 2. Yet, when it comes to distinguishing nodes, H-GSM surpasses IGSM. For example, given the greater value, the distinction between nodes 2 and 1 in H-GSM is obvious.

**Table 1:** Comparison results of simple network node influence evaluation indexes.

Rank	DC	Ks	BC	CC	GSM	IGSM	H-GSM
1	3	3, 1, 2, 0	3	3	3	3	3
2	1, 2	5	1	1, 2	1, 2	2 (13.87)	2 (111.2834)
3	0		2	0	0	1 (13.71)	1 (108.7130)
4	5		0, 4, 5, 6	5	5	0	0
5	4, 6			6	6	5	5
6				4	4	6	6
7						4	4

### Datasets and evaluation criteria

#### Datasets

In this article, we experiment with several unweighted and undirected graphs of varying scales. We examine algorithm performance in terms of running time and influence spread and compare it to that of other algorithms. We apply the Susceptible-Infected-Recovered (SIR) epidemic model as a benchmark simulator over six real networks, including USAir97^36^, Netscience and its largest component subgraph (Netscience1)^37^, Email^38^, Yeast^39^, and Router^40^, in order to compare the performance of the proposed H-GSM with the other indexing methods. Table 2 lists some elementary statistics regarding these networks, including their total number of nodes (*n*), the total number of edges (*m*), maximum and minimum degree (*d*_*max*_ and *d*_*min*_ ), and maximum core value (*core*_*max*_).

**Table 2:** The statistics of six real-world complex networks.

Network	*n*	*m*	$d_{\max } /d_{\min }$	$core_{\max }$
USAir97	332	2126	139 / 1	27
Netscience1	379	914	34 / 1	8
Email	1133	5451	71 / 1	12
Netscience	1461	2742	34 / 1	20
Yeast	2361	7182	66 / 1	10
Router	2113	6632	109 / 1	15

#### SIR model

SIR is a strategy that divides the population into three categories: susceptible (S), infected (I), and recovered (R). Just one node is chosen to be infected in each implementation, while the other nodes are set as vulnerable at each separate run. The seed node infects its neighbors with varying spreading probability $\alpha$,and will recover from the infection with the probabilities $\beta$. Each loop is viewed as a time step *t*, and F(*t*) gives the number of nodes infected at time t, which is used to evaluate the first infected node's effect^41^. When none of the nodes remain diseased, the spreading process comes to an end. The same processes are performed for each node in each network, with 500 iterations.

#### Kendall coefficient

Kendall's coefficient is used to determine how well-simulated rankings by the SIR model match the true rankings reached by centrality measures^42^. Kendall’s coefficient compares the similarity and consistency of two sequences. If the list of ranking strategy corresponds more strongly with the list of rated node-spreading abilities in the SIR model, the ranking method is more successful. The more INs has a larger capacity to propagate. Assume a network comprises n vertices, with $n_{c}$ and $n_{d}$ representing the number of concordant and discordant pairs, respectively. The formula for calculating Kendall's coefficient is as follows:

9 $$\tau = \frac{{n_{c} - n_{d} }}{{0.5n\left( {n - 1} \right)}}$$

The greater the $\tau$ number, the more precise the ranked list generated by the ranking system. In the optimal scenario, $\tau = 1$, the approach and the actual spreading process have identical ranking lists. With a large $\alpha$ value, the spreading would encompass nearly the whole network. In this experiment, the SIR model's spreading probability gradually increases from 0.01 to 0.1.

## Results and discussions

### Computational complexity

We determine how difficult it is to utilise our strategy in order to demonstrate how well it works before discussing how well it works. Computational complexity, often known as algorithm complexity, is the amount of time or space required by an algorithm for a given input size. As previously stated, the method reveals that H-GSM is composed of three major components. Before executing the iSI formula with an $O(time)$ complexity, the first stage determines the DC and Ks values. The second step began with the implementation of Dijkstra's shortest path length, signified by complexity, $O(n^{2} )$ to determine the value of global influence (iGI) based on the values of DC and Ks. After that, the third step is performed to identify INs, which is the multiplication of iSI and iGI. Because our method is an improvement of GSM, we assumed that the overall computing time of H-GSM is also $O(n^{2} )$.

We demonstrate the computational difficulty of our technique by benchmarking its execution against six networks. In terms of execution time, our approach surpasses DC, BC, CC, GSM, and IGSM, as shown in Table 3. Whilst DC is commonly touted to have the simplest form and be the easiest to calculate, in terms of time execution, H-GSM exceeds DC. The techniques in this work are implemented on a Windows 11 platform 64-bit system; the machine hardware configuration is an Intel® Core i7-8550U CPU @ 2.4 Hz processor, 24 GB of RAM; and Python-Visual Studio Code 1.56.2 is used for programming.

**Table 3:** Time execution for computational complexity for six networks.

Network	$t(DC)$	$t(BC)$	$t(CC)$	$t(GSM)$	$t(IGSM)$	$t(H - GSM)$
USAir97	0.6	0.5	0.1	0.3	0.3	0.4
Netscience1	0.6	0.5	0.2	0.4	0.3	0.4
Email	0.5	4.7	1.2	0.4	0.3	0.3
Netscience	0.4	1.6	0.2	0.4	0.4	0.3
Yeast	0.6	17.9	5.5	0.4	0.4	0.4
Router	0.4	14.1	4.4	0.4	0.5	0.3
Average	0.52	6.55	1.93	0.38	0.37	0.35

### Nodes spreading and discrimination comparison

We use the SIR model, which is extensively used in network epidemic dynamics, to quantify node impact contributions by examining their spread and differences across nodes. SIR decides which nodes may spread out faster and wider over time. In general, the importance of a node is proportionate to its capacity to expand. The node with the greatest spreading capacity has the most power.

**Figure 2:**
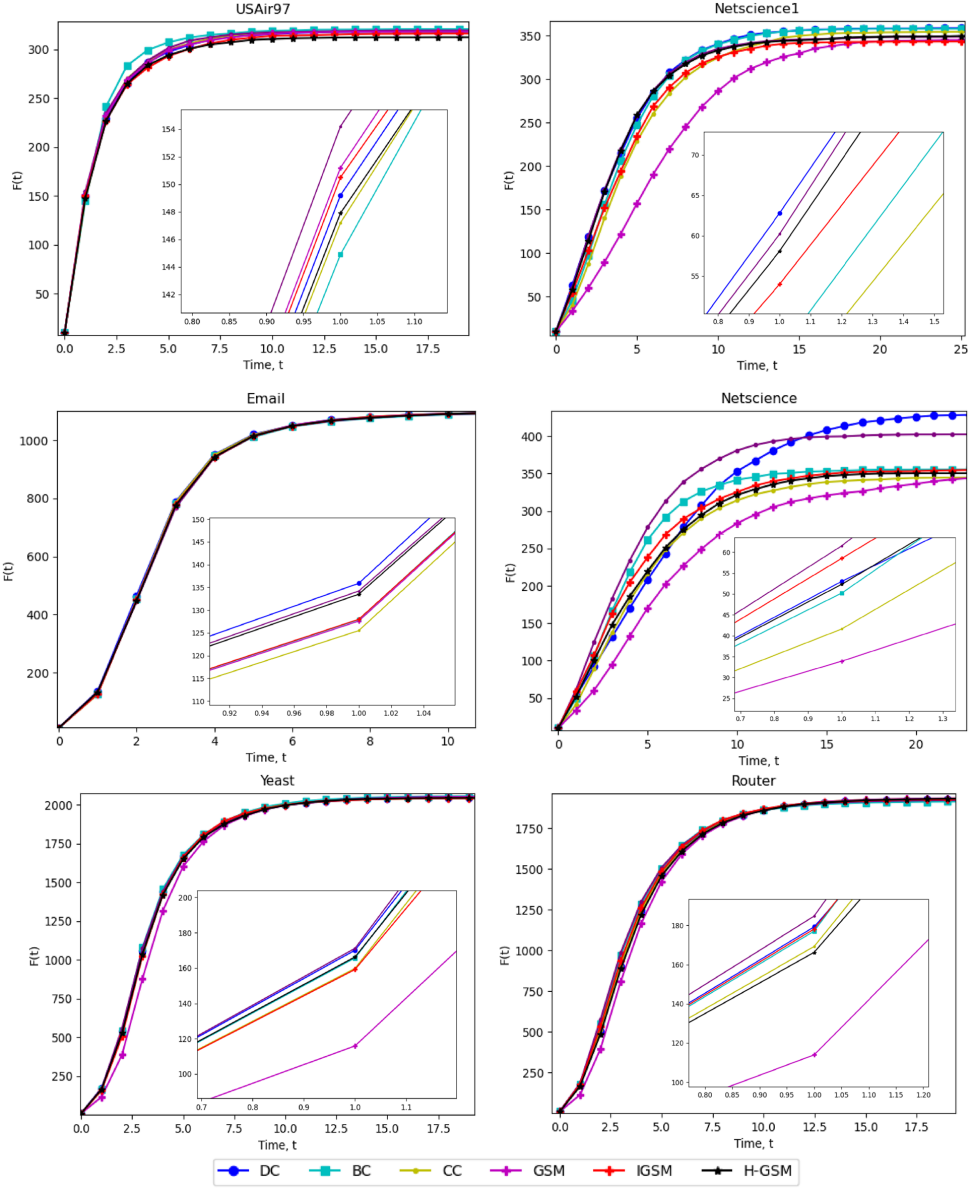
Propagation influence of top 10 nodes of six networks by six methods.

Table 4 shows the top 10 nodes in six networks with great spreading capability using the DC, BC, CC, GSM, IGSM, and H-GSM methodologies. As illustrated in Fig. 2, the ten nodes for each strategy were then used as seed nodes to monitor the nodes' convergence in propagation capacities. The number of infected nodes, F(t), increases with time and quickly stabilises. H-GSM has highly effective propagation throughout the majority of the network.

**Table 4:** Top-10 ranking nodes for: (a) USAir97, (b) Netscience1, (c) Email, (d) Netscience, (e) Yeast and (f) Router network.

Rankings	USAir97						Rankings	Netscience1					
	DC	BC	CC	GSM	IGSM	H-GSM		DC	BC	CC	GSM	IGSM	H-GSM
1	25	25	25	25	25	25	1	3	58	58	4	58	3
2	97	3	97	97	97	97	2	4	106	119	3	4	4
3	95	97	23	95	95	95	3	58	189	106	5	3	58
4	80	27	95	85	85	85	4	5	119	44	13	106	5
5	85	7	27	80	80	80	5	72	72	187	14	119	119
6	90	85	85	58	58	90	6	219	4	107	15	44	13
7	58	95	7	23	90	58	7	119	44	4	16	107	44
8	23	80	28	90	23	23	8	13	187	6	28	5	106
9	24	29	58	27	24	24	9	7	6	130	29	187	107
10	27	10	24	24	27	27	10	106	178	135	119	189	219
(a)							(b)						

Rankings	Email						Rankings	Netscience					
	DC	BC	CC	GSM	IGSM	H-GSM		DC	BC	CC	GSM	IGSM	H-GSM
1	104	332	332	104	104	104	1	43	106	106	44	106	43
2	332	104	22	332	332	332	2	44	184	204	43	44	44
3	15	22	104	22	22	22	3	106	328	184	45	184	106
4	22	577	41	41	41	41	4	45	204	84	53	43	204
5	41	75	40	40	40	40	5	531	120	326	54	204	184
6	40	232	75	75	75	15	6	892	44	185	55	84	84
7	195	134	232	298	232	232	7	893	84	44	56	185	45
8	232	40	51	232	51	195	8	894	326	46	68	326	185
9	20	354	134	51	134	75	9	120	46	215	69	328	53
10	75	41	377	134	377	20	10	143	305	220	204	45	326
(c)							(d)						

Rankings	Yeast						Rankings	Router					
	DC	BC	CC	GSM	IGSM	H-GSM		DC	BC	CC	GSM	IGSM	H-GSM
1	19	446	215	596	215	248	1	100	2	2	89	100	100
2	248	20	35	248	248	581	2	139	0	100	384	139	139
3	441	35	615	581	35	35	3	350	100	89	350	2	350
4	20	19	248	427	20	427	4	62	139	139	356	89	89
5	35	441	20	85	19	19	5	48	159	0	369	0	384
6	446	215	19	279	441	20	6	242	508	242	279	242	0
7	96	248	581	82	581	85	7	113	99	384	381	99	135
8	427	103	441	435	615	441	8	135	350	426	185	62	48
9	581	308	813	250	427	446	9	0	62	99	367	384	2
10	85	427	85	569	85	215	10	51	179	216	100	350	356
(e)							(f)

After that, we analyse if the existence of a node in H-GSM effects its propagation. To analyse H-convergences, GSM's a node from H-GSM that was not existent in GSM is compared with a node from GSM, as shown in Table 4. The following are the findings:

### USAir97

**Figure 3:**
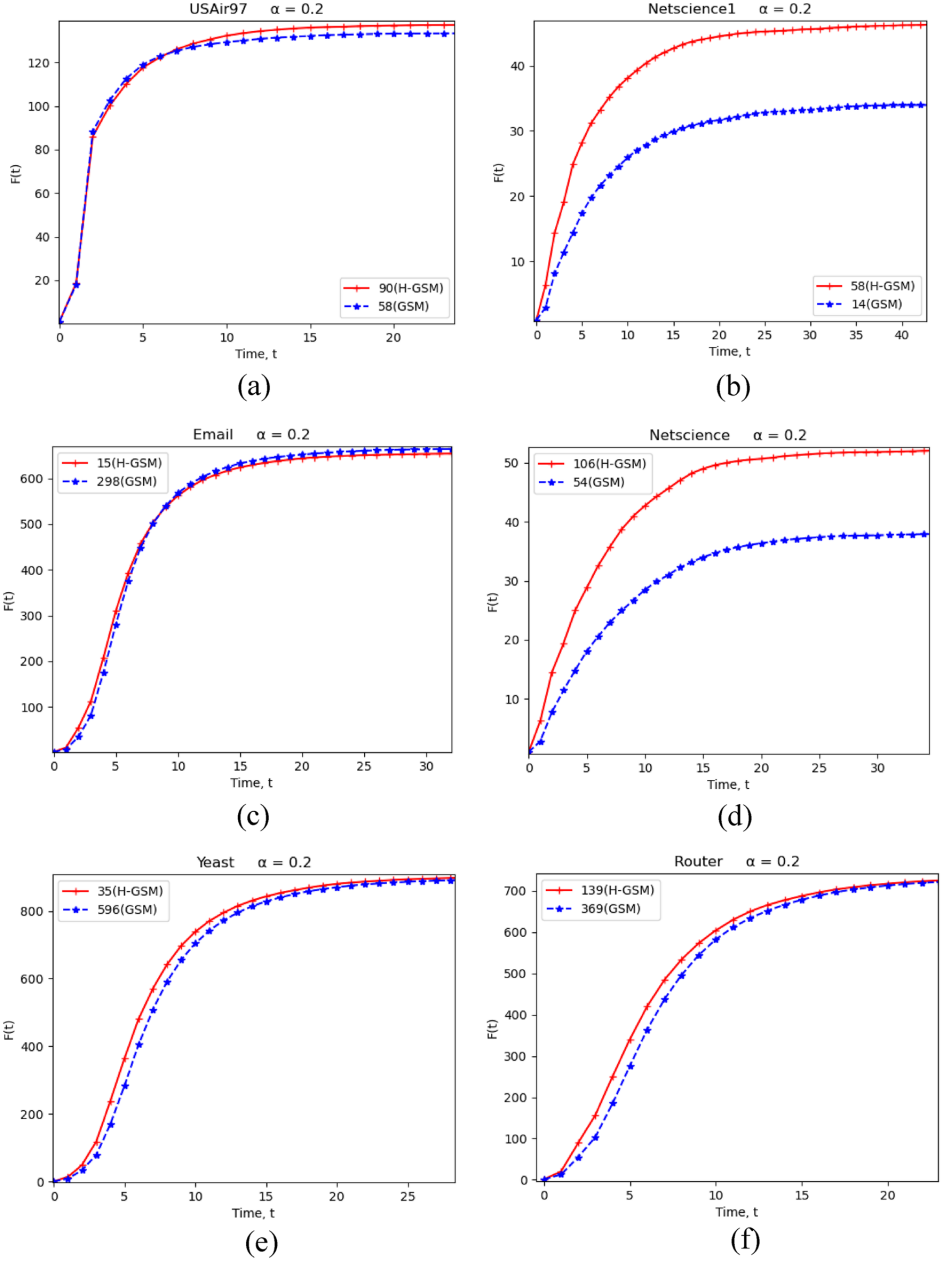
Node's discriminatory comparison of H-GSM and GSM for (**a**) USAir97, (**b**) Netscience1, (**c**) Email, (**d**) Netscience, (**e**) Yeast, and (**f**) Router networks.

All metrics had the same initial affected nodes in terms of node rank. When we looked at GSM, IGSM, and H-GSM, we discovered that their top 10 nodes were identical except for the order. When comparing H-GSM with GSM, the top five nodes are the same, but the sixth varies. As shown in Figure 3a, node 90 (H-GSM) and node 58 (GSM) behave similarly in the beginning, however node 90 had a surge from t = 2 to t = 5. In a while, node 90 may infect others more than node 58 over time.

### Netscience1

The lists produced by the various approaches vary in terms of the rank of each node. The first three lists spanning DC, IGSM, and H-GSM are largely identical, with the main change being the order in which the items occur. When node 58 (H-GSM) is compared to node 14 (GSM), as shown in Figure 3b, we can observe that node 58 performs better since it spreads quicker and further over time.

### Email

It seems that the top five GSM, IGSM, and H-GSM nodes all have similar ranks in this network. The same five nodes appear in all three DC, BC, and CC variations. As illustrated in Figure 3, node 15 (H-GSM) has a quicker node effect than node 298 (GSM), despite their long-term behaviour being very comparable (GSM) (c).

### Netscience

Node 106 appears in the top five node rank lists for every technology except GSM in this network. Hence, if we look at node 106 in H-GSM, we can see that it eventually outperforms node 54 in GSM in terms of how quickly and far it expands over time. Figure 3 displays the results (d).

### Yeast

Similarly, node 35 occurs in the top 10 nodes rated in all methods except GSM. As shown in Figure 3, node 35 (H-GSM) has a greater overall effect than node 596 (GSM) in terms of the pace at which it expands and the total node influence it holds (e).

### Router

The patterns seem to be the same for node 139 (H-GSM) and node 369 (GSM), with the exception that node 139 appears to be more important in that it is spreading faster and broader than node 369. After comparing the rankings for various techniques to the list of node ranks in GSM in Figure 3f, it was discovered that only a handful were enrolled in the top-10 most INs.

### Kendall's model comparison

**Figure 4:**
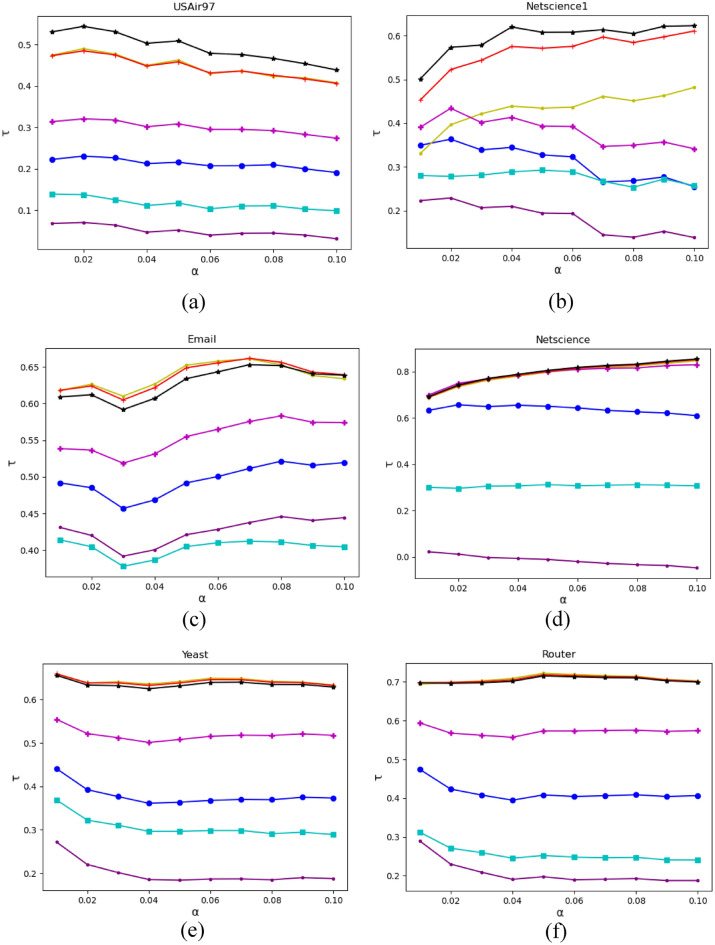
Kendall coefficient results comparisons for the six methods for (**a**) USAir97, (**b**) Netscience1, (**c**) Email, (**d**) Netscience, (**e**) Yeast, and (**f**) Router networks.

The SIR and Kendall models are used to ensure that the relationships discovered between H-GSM and other well-known metrics of centrality are relevant and helpful. Kendall's for the proposed H-GSM and other approaches is shown in Fig. 4. In Kendall's words, H-GSM surpasses state-of-the-art baseline approaches implemented on various network topologies by an alpha of 0.01 to 0.10, proving that it effectively conveys information. The H-GSM surpassed competitors for USAir97 and Netscience 1. H-GSM rates strongly in the Email, Netscience, Yeast, and Router categories while being somewhat inferior to the top two approaches. In comparison to H-GSM and IGSM, the original GSM technique has a low value.

## Conclusion and future recommendations

When H-GSM findings are compared to GSM and other techniques, it is evident that H-GSM is superior. H-GSM is a hybrid strategy that improves on the GSM methodology by taking into account both the network's local and global structure, as represented by the DC and KS approaches, respectively. The capacity of a node to exchange information with all other nodes, as well as the node's own impact, are utilised as indicators to measure the node's influence in the network. The fundamental assumption is that the place and degree of connectedness of nodes are the major drivers of their influence. The more common nodes there are between two nodes, the closer they are, suggesting a better capacity to transfer information. The more significant a neighbour node is, the more it contributes to a node's influence.

To analyse the usefulness of the proposed strategy, we use the SIR model to simulate the propagation process and perform tests on the two major aspects of discrimination and accuracy in diverse real-world networks. First, when compared to other techniques, H-GSM has the lowest average computational complexity in terms of execution time. Second, the proposed technique illustrates that integrating local and global information may successfully decouple a node's value by comparing the top ten most INs. Moreover, the suggested technique in the research outperforms others in ranking correlation, proving its high accuracy. Finally, by integrating the strengths of the DC and Ks indices and incorporating additional self- and global impact metrics, the proposed H-GSM algorithm improves on previous techniques. The addition of location and node data improves its capacity to recognise important nodes in a network. We think that our suggested technique will make a substantial contribution to the area of network analysis and will be beneficial in a variety of applications.

In conclusion, this paper has presented the H-GSM as a novel approach for analyzing complex networks. By considering both local and global information of each node, the H-GSM algorithm addresses the deficiencies of current techniques and offers a more comprehensive understanding of network structures. H-GSM algorithm presents a significant step forward in network analysis, enabling researchers to gain deeper insights into complex network structures and identify INs with improved accuracy and scalability. This is one of our early efforts by using the network's topological connection structure. Additionally, we will continue our study by evaluating other combinations and validation approaches in order to increase the performance of the methodologies offered.

## Data Availability

The dataset that being used in this article can be downloaded freely from These networks can be downloaded from KONECT (http://konect.cc/networks/) and NETWORK (https://networkrepository.com/networks.php).

## References

[1] Ramli, M. R., Hussin, B., Abas, Z. A. & Ibrahim, N. K. Solving complex nurse scheduling problems using particle swarm optimization. *Int. Rev. Comput. Softw.* **11**(9), 834. 10.15866/irecos.v11i9.9881 (2016).

[2] Anuar, S. H. H. *et al.* Comparison between Louvain and Leiden algorithm for network structure: a review. *J. Phys.: Conf. Ser.* **2129**, 012028 (2021).

[3] Abas, Z. A. *et al.* Analytics: A review of current trends, future. *Compusoft.* **9** (2020).

[4] Wang, Y., Wang, S. & Deng, Y. A modified efficiency centrality to identify influential nodes in weighted networks. *Pramana J. Phys.* **92** (2019).

[5] Wang S, Du Y, Deng Y (2017). A new measure of identifying influential nodes: Efficiency centrality. Commun. Nonlinear Sci. Numer. Simul..

[6] Oldham, S. *et al.* Consistency and differences between centrality measures across distinct classes of networks. *PLoS One* **14** (2019). 10.1371/journal.pone.0220061 PMC6660088 31348798

[7] Mukhtar, M. F. *et al.* Identifying influential nodes with centrality indices combinations using symbolic regressions. *Int. J. Adv. Comput. Sci. Appl.* **13** (2022).

[8] Vignery, K. & Laurier, W. A methodology and theoretical taxonomy for centrality measures: What are the best centrality indicators for student networks? *PLoS One* **15** (2020). 10.1371/journal.pone.0244377 PMC7773201 33378341

[9] Jalili M (2015). CentiServer: A comprehensive resource, web-based application and R package for centrality analysis. PLoS ONE.

[10] Pittel, B., Spencer, J. & Wormald, N. Sudden emergence of a giant k-core in a random graph. *J. Combin. Theory Ser. B* **67** (1996).

[11] HamaKarim BR, Mohammadiani RP, Sheikhahmadi A, Hamakarim BR, Bahrami M (2023). A method based on k-shell decomposition to identify influential nodes in complex networks. J. Supercomput..

[12] Freeman, L. C. Centrality in social networks conceptual clarification. *Soc. Netw.* **1** (1978).

[13] Liu, Y., Tang, M., Zhou, T. & Do, Y. Improving the accuracy of the k-shell method by removing redundant links: From a perspective of spreading dynamics. *Sci Rep* **5** (2015). 10.1038/srep13172 PMC4538382 26277903

[14] Barabási AL (2013). Network science. Philos. Trans. R. Soc. A Math. Phys. Eng. Sci..

[15] Wang, F. *et al.* Influential node identification by aggregating local structure information. *Physica A Stat. Mech. Appl.* **593** (2022).

[16] Garas, A., Schweitzer, F. & Havlin, S. A κ-shell decomposition method for weighted networks. *New J. Phys.* **14** (2012).

[17] Shetty RD, Bhattacharjee S, Dutta A, Namtirtha A (2022). GSI: An influential node detection approach in heterogeneous network using covid-19 as use case. IEEE Trans. Comput. Soc. Syst..

[18] Zhu, J. C. & Wang, L. W. An extended improved global structure model for influential node identification in complex networks. *Chin. Phys. B* **31** (2022).

[19] Lü, L., Zhou, T., Zhang, Q. M. & Stanley, H. E. The H-index of a network node and its relation to degree and coreness. *Nat. Commun.* **7** (2016). 10.1038/ncomms10168 PMC4729922 26754161

[20] Newman, M. *Networks: An Introduction*. *Networks: An Introduction* (2010). 10.1093/acprof:oso/9780199206650.001.0001.

[21] Brandes, U. & Erlebach, T. *Network Analysis: Methodological Foundations*. *Lecture Notes in Computer Science* vol. 3418 (2005).

[22] Scott, J., Wasserman, S., Faust, K. & Galaskiewicz, J. Social network analysis: methods and applications. *Br. J. Sociol.* **47**, (1996).

[23] Borgatti SP (2005). Centrality and network flow. Soc. Netw..

[24] Freeman, L. C. A set of measures of centrality based on betweenness. *Sociometry* **40** (1977).

[25] Bavelas, A. Communication patterns in task-oriented groups. *J. Acoust. Soc. Am.* **22** (1950).

[26] Ibnoulouafi, A., el Haziti, M. & Cherifi, H. M-Centrality: Identifying key nodes based on global position and local degree variation. *J. Stat. Mech. Theory Exp.* **2018** (2018).

[27] Al-garadi MA, Varathan KD, Ravana SD (2017). Identification of influential spreaders in online social networks using interaction weighted K-core decomposition method. Physica A.

[28] Berberler ME (2020). Global and local structure-based influential nodes identification in wheel-type networks. Numer. Methods Partial Differ. Equ..

[29] Ullah, A. *et al.* Identification of nodes influence based on global structure model in complex networks. *Sci. Rep.* **11** (2021). 10.1038/s41598-021-84684-x PMC7969936 33731720

[30] Sheng, J. *et al.* Identifying influential nodes in complex networks based on global and local structure. *Physica A Stat. Mech. Appl.* **541** (2020).

[31] Gouveia, C., Móréh, Á. & Jordán, F. Combining centrality indices: Maximizing the predictability of keystone species in food webs. *Ecol. Indic* **126** (2021).

[32] Zareie A, Sheikhahmadi A (2019). EHC: Extended H-index Centrality measure for identification of users’ spreading influence in complex networks. Physica A.

[33] Curado, M., Tortosa, L. & Vicent, J. F. A novel measure to identify influential nodes: Return Random Walk Gravity Centrality. *Inf. Sci. (NY)* **628** (2023).

[34] Fariduddin Mukhtar, M. *et al.* Global structure model modification to improve influential node detection. **18** (2023).

[35] Talib, Mohammed Saad, *et al.* "Clustering based affinity propagation in VANETs: Taxonomy and opportunity of research." *Int. J. Recent Technol. Eng* **7**, 6S5, 672–679 (2019).

[36] Rossi, R. A. & Ahmed, N. K. The network data repository with interactive graph analytics and visualization. In *Proceedings of the National Conference on Artificial Intelligence* vol. 6 (2015).

[37] Newman, M. E. J. Finding community structure in networks using the eigenvectors of matrices. *Phys. Rev. E Stat. Nonlin. Soft Matter. Phys.* **74** (2006). 10.1103/PhysRevE.74.036104 17025705

[38] Vanderstraeten, L., Vanhecke, B. & Verstraete, F. Residual entropies for three-dimensional frustrated spin systems with tensor networks. *Phys. Rev. E* **98** (2018).

[39] Bu, D. *et al.* Topological structure analysis of the protein-protein interaction network in budding yeast. *Nucleic Acids Res.* **31** (2003). 10.1093/nar/gkg340 PMC154226 12711690

[40] Spring, N., Mahajan, R., Wetherall, D. & Anderson, T. Measuring ISP topologies with rocketfuel. *IEEE/ACM Trans. Network.* **12** (2004).

[41] Yang, P., Liu, X. & Xu, G. A dynamic weighted TOPSIS method for identifying influential nodes in complex networks. *Modern Phys. Lett. B* **32**, (2018).

[42] Kendall, M. G. A New Measure of Rank Correlation. *Biometrika* **30**, (1938).

